# An extraordinary rare anastomotic band causing food bolus obstruction following uneventful minimally invasive esophagectomy: endoscopic treatment

**DOI:** 10.1093/jscr/rjab212

**Published:** 2021-05-27

**Authors:** Efstratia Baili, Spyridon Davakis, Athanasios Syllaios, Maria Boura, Antonia Meropouli, Alexandros Charalabopoulos

**Affiliations:** 1st Department of Surgery, Laiko General Hospital, National and Kapodistrian University of Athens, Athens 11527, Greece; 1st Department of Surgery, Laiko General Hospital, National and Kapodistrian University of Athens, Athens 11527, Greece; 1st Department of Surgery, Laiko General Hospital, National and Kapodistrian University of Athens, Athens 11527, Greece; 1st Department of Surgery, Laiko General Hospital, National and Kapodistrian University of Athens, Athens 11527, Greece; 1st Department of Surgery, Laiko General Hospital, National and Kapodistrian University of Athens, Athens 11527, Greece; 1st Department of Surgery, Laiko General Hospital, National and Kapodistrian University of Athens, Athens 11527, Greece

**Keywords:** Post-esophagectomy syndromes, Esophageal strictures, Minimally invasive, Endoscopy, Esophageal stenosis

## Abstract

The most common long-term complication post esophagectomy implicating the esophagogastric anastomosis is stricture-induced stenosis leading to late postoperative dysphagia. Herein, we present a case of a male patient readmitted to our Upper Gastrointestinal Department due to a food bolus obstruction through an anastomotic epithelial band arisen from a prior esophagogastric anastomosis performed 5 months earlier. A band transection in between two hemostatic clips placed on both sides of the band, followed by a release and fragmentation of the foreign body into several pieces led to its final transoral removal endoscopically. The patient experienced a direct resolution of his dysphagia and discharged on the same day. At 6 months follow-up, he remains symptom-free. In conclusion, endoscopic state-of-the-art techniques when combined with standard hemostatic surgical principles in a minimally invasive manner are excellent tools for the management of post-esophagectomy syndromes.

## INTRODUCTION

The most frequent late complication post-esophagectomy implicating the esophagogastric anastomosis is stricture-induced stenosis and associated dysphagia [1]. Anastomotic strictures can be further classified into either scar contractures or anastomotic leakage-induced strictures [2]. Herein, we report a case of a 70-year-old Caucasian male patient readmitted to our Upper Gastrointestinal Department due to dysphagia not related to stricture following an uneventful oncological minimally invasive esophagectomy conducted 5 months earlier.

## CASE REPORT

### Background

Patient’s medical history started 1 year ago, when he was submitted to a minimally invasive esophagectomy due to a Siewert II adenocarcinoma of the gastroesophageal junction. His postoperative course was unremarkable and discharged on the 8th postoperative day in a good overall condition. The final histological examination of the specimen was conclusive of an adenocarcinoma of the gastroesophageal junction, pT1N0Mx, with adequate resection margins. At 3 months follow-up, the patient presented well, experiencing no dysphagia or vomiting symptoms.

However, 2 months later he presented to our institution with acute dysphagia to both liquids and solids, deteriorating over the past week. While subsequent work-up with computed tomography and barium esophagram displayed neither disease progression nor anastomotic stenosis, the endoscopic examination revealed the presence of a bulky food particle wedged at the anastomotic level due to an intraluminal epithelial anastomotic band. This is the first ever-reported case of post-esophagectomy dysphagia related to an intraluminal epithelial band across the esophagogastric anastomosis.

#### Endoscopic technique and technologies

**Figure 1:**
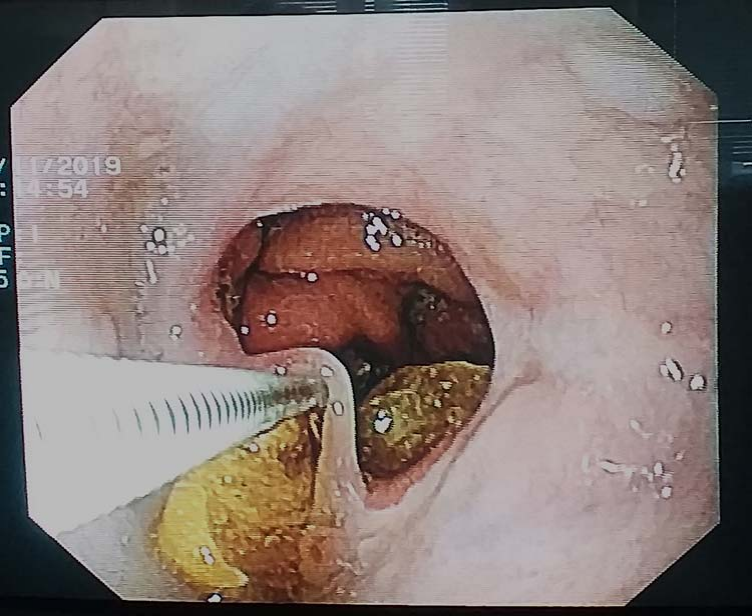
Endoscopic appearance of the bulky food particle impacted around the epithelial band arisen from the esophagogastric anastomosis.

**Figure 2:**
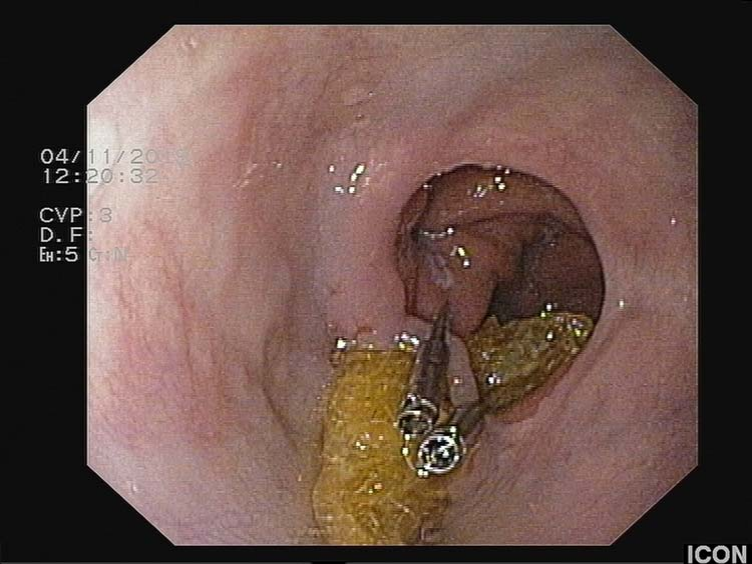
Placement of two hemoclips on both sides of the anastomotic band prior to transection, in a scope’s straight position.

**Figure 3:**
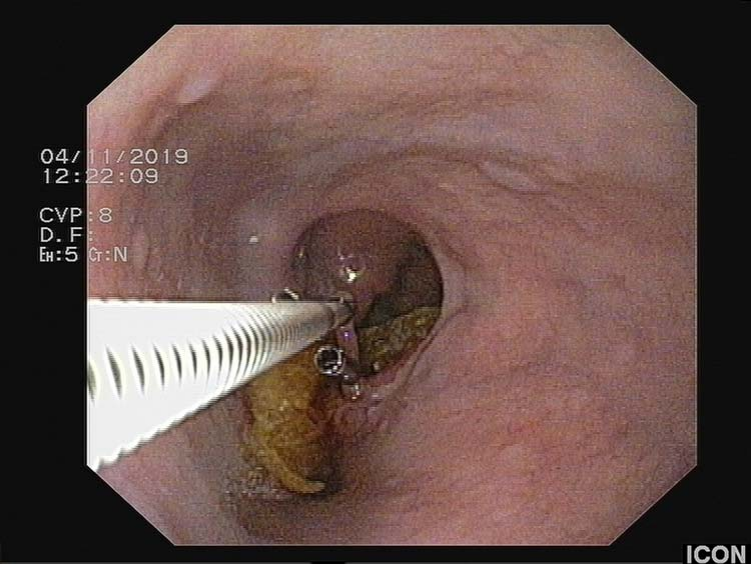
Flexible endoscopic scissors transecting the epithelial band in between the hemostatic clips.

**Figure 4:**
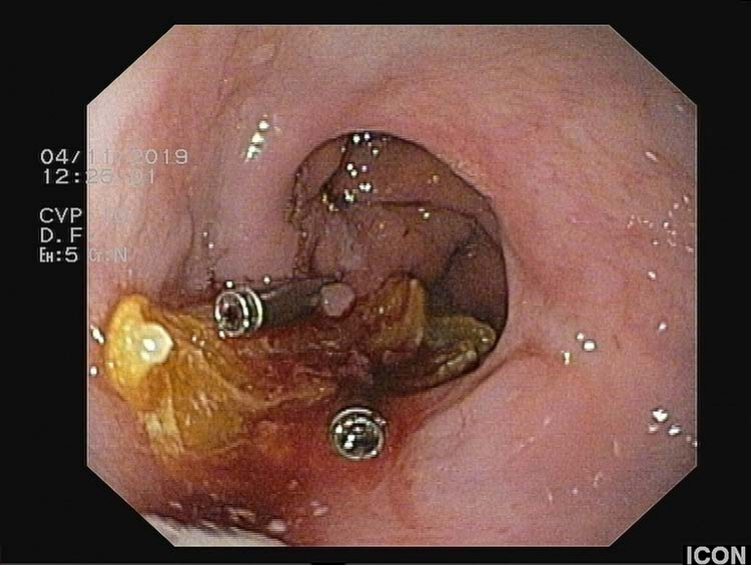
The large food material is released from the esophageal lumen with no blood loss.

**Figure 5:**
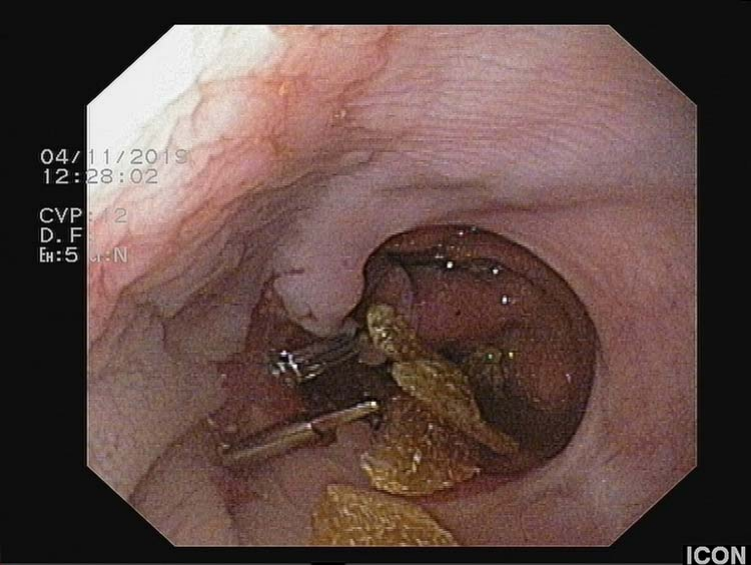
The foreign body is fragmented with grasping forceps to facilitate transoral removal.

A Karl-Storz Flexible Routine Interventional Gastroscope with Two Working/Suction Channels (Karl-Storz SE & Co. KG Tuttlingen, Germany) with an outer diameter of 9.3 mm and a working length of 1100 mm was used. The large food particle was detected impacted between the anastomotic epithelial band and the anastomotic wall with the distal end protruding freely into the gastric conduit lumen and the proximal end into the esophageal lumen, 33 cm far from the incisors (Fig. 1). Several successive attempts with grasping forceps to dislodge it from the esophagus were unsuccessful. With respect to patient’s good overall status with no coagulation disorders, an alternative approach was implemented under conscious sedation. Upon recognition of the epithelial band, two hemostatic clips were deployed on its both sides for hemostasis, in a scope’s straight position (Fig. 2). This was followed by band transection in between the two clips with endoscopic scissors, release of the foreign body, subsequent fragmentation of it into several pieces and finally, its transoral removal (Figs 3–5, Supplemental File).

#### Outcomes

Procedural time was 20 min, estimated blood loss was <20 ml, with no periprocedural complications. Two hours post-intervention, the patient was subjected to barium swallow examination that demonstrated no contrast extravasation. He was then allowed a semi-solid diet and discharged on the same day, with direct resolution of his dysphagia. At 6 months follow-up post endoscopic intervention, he remains symptom-free.

## DISCUSSION

The most common long-term complication post esophagectomy implicating the esophagogastric anastomosis is stricture-related stenosis and associated dysphagia with an incidence of 5–40% [3]. The most valuable investigation tool is an upper gastrointestinal endoscopy with utilization of endoscopic dilatation of anastomotic strictures, stent placement or endoscopic incision [4, 5]. The rate of successful endoscopic interventions has notably increased over time contributing to a significant decline in the need for surgical reintervention, nowadays limited just for the refractory cases [6].

To the best of our knowledge, the present case with food bolus obstruction due to an intraluminal epithelial anastomotic band is the first ever reported case of post-esophagectomy dysphagia—not induced by a stricture—in the literature. Several important technical principles are highlighted. An optimal level of suspicion accompanied by a sufficient knowledge in the field of post-esophagectomy complications is compulsory for the early detection of such entities. The introduction of minimally invasive techniques along with the rapid improvement of endoscopic skills has revolutionized esophageal surgery by successfully managing the majority of surgical postoperative complications, avoiding surgical exploration and associated trauma, reducing hospitalization, and by enhancing postoperative recovery [7].

The present report demonstrates that endoscopic state-of-the-art techniques when combined with standard hemostatic surgical principles in a minimally invasive manner are excellent tools for the management of post-esophagectomy syndromes overall [8, 9].

## Supplementary Material

Food_bolus_obstruction_post_esophagectomy_rjab212
